# Onychomadesis Developed Only on the Nails Having Cutaneous Lesions of Severe Hand-Foot-Mouth Disease

**DOI:** 10.1155/2011/324193

**Published:** 2011-12-27

**Authors:** Emi Shikuma, Yuichiro Endo, Akihiro Fujisawa, Miki Tanioka, Yoshiki Miyachi

**Affiliations:** Department of Dermatology, Kyoto University, 54 Shogoin Kawaramachi Sakyo, Kyoto 606-8507, Japan

## Abstract

This paper reported a case of onychomadesis which appeared on the nails after heal of cutaneous lesions of hand-foot-mouth disease (HFMD). There were a few reports describing onychomadesis after HFMD; however, the mechanism is still unclear. The present case was prospectively observed, and onychomadesis was found to develop only on the nails having cutaneous lesions of HFMD. We considered that nail dysfunction due to direct inflammation spreading from skin eruptions around nail is one of the causes of onychomadesis linked to *HFMD*.

## 1. Introduction

Hand-foot-mouth disease (HFMD) is an acute virus infectious disease that becomes common among children in summer. The most common strains causing HFMD are Coxsackie A virus and Enterovirus 71. Recently, a few reports described onychomadesis after HFMD; however, the mechanism is still unclear. Onychomadesis is a periodic idiopathic shedding of the nails beginning at its proximal end, possibly caused by the temporary arrest of the function of the nail matrix. Here, we presented a case of onychomadesis, which developed only on the nails having cutaneous lesions of HFMD.

## 2. Case Presentation

**Figure 1:**
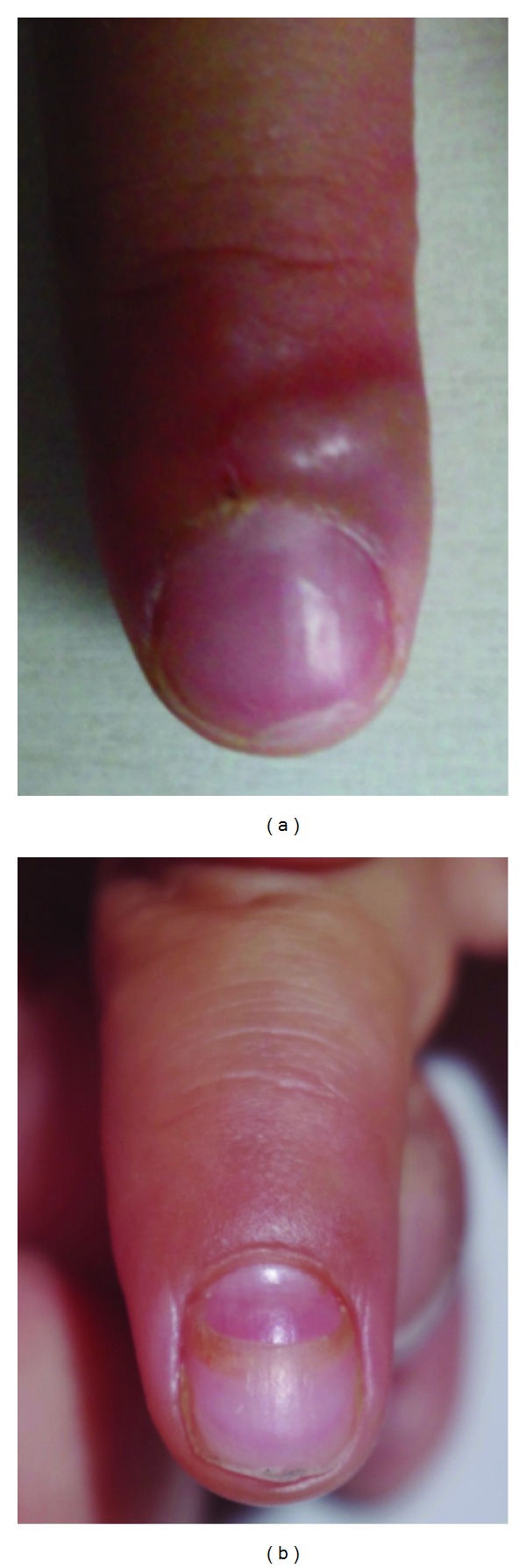
(a) A large pustule existed around the nail of the right index finger, looking like a herpetic whitlow. (b) At four weeks after the disappearance of cutaneous lesions of HFMD, onychomadesis appeared in the nail of the right index finger.

A 5-year-old boy presented with oval vesicles on the hands, feet, and mouth. The typical cutaneous lesions led us to the diagnosis of HFMD. On the right index finger, a large pustule existed around the nail, looking like a herpetic whitlow (Figure 1(a)). The cutaneous lesions around nails were observed on the left index and middle fingers and bilateral thumbs and 1st toes. He had no fever and was generally in a good condition. He took no medication during the course. The skin lesions naturally healed. A dermatologist (author: M. Tanioka) prospectively observed all nails. At four weeks after the disappearance of cutaneous lesions of HFMD, onychomadesis appeared in the nails of the same fingers and toes (Figure 1(b)). The nail changes were temporary with spontaneous normal regrowth.

## 3. Discussion

Onychomadesis is an acute, painless, noninflammatory disease that affects the nail matrix. In 2000, the first description of onychomadesis following HFMD was reported in USA [1]. Onychomadesis accompanying outbreaks of coxsackievirus type A6 (CA6) has been reported in Spain and Finland, though onychomadesis could be caused by the different enterovirus serotypes [2, 3]. Patients present a wide clinical profile from transverse ridging of the nail plate (Beau's lines) up to complete nail shedding. Apart from serious generalised diseases, trauma, or exposure to specific drugs, most cases have been considered idiopathic. Also in Japan, an outbreak of HFMD due to CA6 is occurring in 2011. The characteristic features of HFMD in 2011 in Japan are onychomadesis, adult cases with severe systemic symptoms, larger skin eruptions, wider distributions on the face and buttocks besides the hands and feet. Onychomadesis might be one of the characteristics of HFMD due to coxsackievirus type A6.

 The mechanism of onychomadesis is still unknown; however, onychomadesis means that nail matrix proliferation was temporarily inhibited. It is under discussion whether the inhibition resulted from direct inflammation spreading from skin lesions of HFMD around nails or coxsackievirus-specific nail dysfunction, or HFMD's severe systemic impact on the general condition of the small children [4]. Reported onychomadesis without preceding skin eruptions around nails suggested that it was coxsackievirus-specific nail dysfunction. However, all previous reports describing onychomadesis were retrospectively analyzed. In this paper, all nails were prospectively observed and onychomadesis corresponded with fingers and toes having severe cutaneous eruptions around nails. At least, nail dysfunction due to direct inflammation spreading from skin eruptions around nail is one of the causes of onychomadesis linked to HFMD.
